# The Regulatory Mechanism of Feeding a Diet High in Rice Grain on the Growth and microRNA Expression Profiles of the Spleen, Taking Goats as an Artiodactyl Model

**DOI:** 10.3390/biology10090832

**Published:** 2021-08-26

**Authors:** Qiongxian Yan, Kaijun Wang, Xuefeng Han, Zhiliang Tan

**Affiliations:** 1 CAS Key Laboratory of Agro-Ecological Processes in Subtropical Region, Hunan Provincial Key Laboratory of Animal Nutritional Physiology and Metabolic Process, Institute of Subtropical Agriculture, Chinese Academy of Sciences, Changsha 410125, China; yanqx14@isa.ac.cn (Q.Y.); kj-wang@alu.gxu.edu.cn (K.W.); xfhan@isa.ac.cn (X.H.); 2 Hunan Co-Innovation Center of Animal Production Safety—CICAPS, Changsha 410128, China

**Keywords:** rice grain, microRNA, LPS, spleen, goats

## Abstract

**Simple Summary:**

Feeding with a diet high in rice often results in several adverse effects for animals, such as ruminal acidosis, the translocation of endotoxin into the bloodstream, gastrointestinal barrier impairment, liver injures, and inflammation. A high rice grain-based diet also provides excess energy for human beings, which usually induces obesity via an miRNA-based mechanism. Currently, whether high-rice diets affect spleen growth and related molecular events remains unknown. Therefore, we take goats as an artiodactyl model to investigate the effects of feeding a highly concentrated diet, based on rice grains, on the growth and microRNA expression profiles in the goat spleen. Here, we first report about the splenic microRNA profile in artiodactyls fed a highly concentrated diet based on rice. Our results shed light on the miRNA-based regulatory mechanism that contributes to the development of high calorie-induced metabolic diseases in mammals.

**Abstract:**

Several researchers have testified that feeding with diets high in rice grain induces subacute ruminal acidosis and increases the risk of gastrointestinal inflammation. However, whether diets high in rice grain affect spleen growth and related molecular events remains unknown. Therefore, the present study was conducted to investigate the effects of feeding a high-concentrate (HC) diet based on rice on the growth and microRNA expression profiles in goat spleen. Sixteen Liuyang black goats were used as an artiodactyl model and fed an HC diet for five weeks. Visceral organ weight, LPS (lipopolysaccharide) concentration in the liver and spleen, and microRNA expression were analyzed. The results showed that feeding an HC diet increased the heart and spleen indexes and decreased the liver LPS concentration (*p* < 0.05). In total, 596 microRNAs were identified, and twenty-one of them were differentially expressed in the spleens of goats fed with the HC diet. Specifically, several microRNAs (miR-107, miR-512, miR-51b, miR-191, miR-296, miR-326, miR-6123 and miR-433) were upregulated. Meanwhile, miR-30b, miR-30d, miR-1468, miR-502a, miR-145, miR-139, miR-2284f, miR-101 and miR-92a were downregulated. Additionally, their target gene *CPPED1*, *CDK6*, *CCNT1* and *CASP7* expressions were inhibited (*p* < 0.05). These results indicated that the HC diet promoted the growth of the heart and spleen. The HC diet also regulated the expression of miR-326, miR-512-3p, miR-30b, miR-30d, miR-502a and their target genes (*CPPED1*, *CDK6* and *CCNT1*) related to the enhancement of splenocyte proliferation. The HC diet also modulated the expression of miR-15b-5p, miR-1468 and miR-92a, related to the suppression of splenocyte apoptosis.

## 1. Introduction

As the largest secondary lymphoid organ, the spleen affects an extensive range of immunological functions in the bodies of mammals. Based on its anatomical structure, the spleen can be divided into red pulp (RP), white pulp (WP), and the marginal zone that is located between the RP and WP. The RP filters out aged red blood cells from the circulatory system, while simultaneously surveying for pathogen and tissue damage. Immune effector function, extramedullary hematopoiesis, and the storage of cellular reserves take place in the RP [1,2]. As for the WP, adaptive immune responses to systemic antigens are often launched from here. It functions as the secondary lymphoid organ of the circulatory system, draining and monitoring antigens from tissues. Naive and central memory T cells residing in the WP are activated by cognate antigen stimulation and T-dependent B cell germinal center reactions, resulting in antibody production [3]. The marginal zone is an important entrance where lymphocytes are released from the circulation to the WP. Certain types of B cells or macrophages reside in this region in a steady state.

The microRNAs are endogenous short single-stranded RNA molecules, and act as key regulators of gene expression by directly binding to the 3′-untranslated region of target mRNAs, resulting in the degradation of the target mRNA [4,5]. By mediating target-gene expression, microRNAs have been implicated in triggering inflammation and tumorigenesis, as well as regulating important biological functions, such as immune cell differentiation, development, and immune response [6]. In an acute *Toxoplasma gondii* infection, a changed microRNAs regulation network in the mouse spleen has been observed [7]. The functional deletion of a single microRNA also alters the composition of immune cells [8] and T lymphocyte function in the spleens of mice [9]. These in vivo and in vitro experiments demonstrate that microRNAs are also involved in parasite-host interaction and the immune response of the spleen.

Excess calorie supply, such as a high-fat diet, usually induces obesity via an miRNA-based mechanism, with the observation of a reduction in miR-155 and miR-146a expressions within specific brain nuclei in mice and unchanged expression in peripheral tissue, including the spleen [10]. However, caloric restriction is well known to prolong median and maximal lifespans and to reduce the mortality and morbidity of short-lived animal models. Specifically, miR-16-5p expression can be enhanced in the spleen, thymus, colon and stomach of caloric-restricted mice and might be involved in regulating aging and longevity [11]. Multiple studies have reported that feeding with a high-corn diet with excess energy supply is a favorite measure to support rapid weight gain at the finishing stage of artiodactyls. However, this feeding practice has brought several adverse effects, such as ruminal acidosis [12], the translocation of endotoxin into the bloodstream [13], gastrointestinal barrier impairment [14,15], liver injures, and inflammation [16]. However, corn is relatively scarce in the southern area of Asia. Rice crops are widely planted in this region, becoming an alternative feedstuff applied to animal feeding. Our research group has demonstrated that a high-concentrate diet (HC) based on rice grains also causes subacute ruminal acidosis [17], and changes the ileal microbial community and gut inflammation [18] of growing goats. Therefore, we continued to use goats as an artiodactyls model to figure out whether this kind of HC diet would affect the spleen growth, microRNA profile and microRNA expression of the target genes. The findings of this study will shed light on the miRNA-based regulatory mechanism, contributing to the understanding of high calorie-induced metabolic diseases in mammals.

## 2. Materials and Methods

### 2.1. Experimental Design and Animal Management

Sixteen Liuyang black goats with a similar body weight of 20.2 ± 1.5 kg were randomly allocated to two treatment groups: normal-concentrate diet (NC, *n* = 8) and high-concentrate diet (HC, *n* = 8). Goats in the NC group were fed a diet with a ratio of concentrate to forage of 55:45, while goats in the HC group received a diet with a ratio of concentrate to forage of 90:10. The rice straw was selected as forage and was chopped to approximately 2 cm in length before feeding. The ingredients and nutritional levels of the experimental diets are listed in Table 1. The experimental period consisted of 35 days, with seven days for diet adaptation and four weeks for sampling. Diets were equally offered at 08:00 and 18:00 daily, respectively. Each experimental goat was fed in a separate cage and had free access to water. The feed intake of each goat was recorded every day.

### 2.2. Sample Collection

On day 35, blood samples were collected aseptically into tubes with heparin sodium from the jugular vein after 24 h of fasting. Plasma was separated by centrifugation at 1000× *g* for 15 min at 4 °C and stored at −20 °C. After euthanizing with an intravenous injection of sodium pentobarbital (50 mg/kg BW), the visceral organs (including the heart, liver, spleen, lung and kidney) were collected and weighed immediately. Liver tissues were washed with 0.9% (*w/v*) ice-cold saline, cut into small pieces, and stored at −20 °C. Spleen tissues were collected about 1 g from the outer edge (RP), avoiding blood vessels. Then, spleen tissues were washed with 0.9% (*w/v*) ice-cold saline, cut into small pieces, snap-frozen in liquid nitrogen and stored at −80 °C.

### 2.3. LPS Determination

LPS concentration in blood, liver and spleen was assayed using the goat ELISA kit according to the manufacturer’s instructions (MEIMIAN, Jiangyin, China). The intra-assay and inter-assay precision CV% are less than 10% and the minimum detectable dose is typically less than 1.0 ng/mL.

### 2.4. RNA Isolation, Small RNA Library Construction, and Sequencing

Total RNA from the spleen tissues was extracted using a TRK-1002 total RNA purification kit (LC Sciences). Total RNA quantity and integrity were assessed according to Zhang et al. (2019) [19]. Approximately 1 μg of total RNA from each sample was used for library preparation. Then, ten small libraries (four samples from the NC group and six samples from the HC group) were used for Illumina/Solexa deep sequencing. Ligation of the total short RNAs (~18–26 nt in length), reverse transcription and PCR were performed according to previous work [20]. Finally, the amplification products were used directly for cluster generation and were then submitted to LC-Bio (Hangzhou, China) for single-end sequencing on an Illumina Hiseq2500.

### 2.5. Bioinformatics Analysis of Solexa Sequencing Data

Raw reads were subjected to the ACGT101-miR program (version 4.2, LC Sciences, Houston, TX, USA) to remove adapter dimers, junk, low complexity, common RNA families including rRNA, tRNA, snRNA and snoRNA, and repeats. Then, unique sequences with a length in the range of 18~26 nucleotides (nt) were matched to *Capra hircus* (CHIR_2.0) precursors in the miRBase 21.0 database. The unique sequences mapping to specific species of mature miRNAs in hairpin arms were identified as known miRNAs. The unique sequences mapping to the other arm of a known species-specific precursor hairpin opposite to the annotated mature miRNA-containing arm were considered to be novel 5p- or 3p-derived miRNA candidates. The unmapped sequences were BLASTed against the specific genomes, and the hairpin RNA structures containing the sequences were predicated from the flanking 80 nt sequences using the RNAfold software (http://rna.tbi.univie.ac.at/cgi-bin/RNAfold.cgi, accessed on 16 May 2019). A modified global normalization method is applied to correct copy numbers of miRNAs among ten spleen samples. The basic assumptions and procedures involved have been described before in [21].

The differential expression of miRNAs (DEmiRNAs) based on normalized deep-sequencing counts was analyzed by selectively using a Student’s *t*-test. Both a *p*-value less than 0.05 and the absolute value of the log_2_ fold change (HC/NC) ≥ 1 were defined as DEmiRNA. The heatmap of DEmiRNAs was drawn using the Pheatmap package (Version: 1.0.12). When predicting the genes targeted by DEmiRNAs, both algorithms in TargetScan 5.0 (http://www.targetscan.org/vert_50/ accessed on 16 June 2011) and Miranda 3.3a (http://cbio.mskcc.org/microrna_data/miRanda-aug2010.tar.gz accessed on 16 August 2010) were adopted to identify the miRNA binding sites. Then, the data were combined, and the overlaps were calculated. The GO enrichment analysis and pathway enrichment analysis of differentially expressed miRNA (DEmiRNAs) target genes were performed by mapping to GO terms in the gene ontology database (http://www.geneontology.org/ accessed on 16 May 2019) and comparing these genes with the whole genome background in the KEGG database (http://www.genome.jp/kegg/ accessed on 16 May 2019), respectively. Gene numbers were calculated for every term or pathway; significantly enriched GO terms or pathways in DEmiRNAs target genes compared to the genome background were defined by a hypergeometric test. The calculating formula for the *p*-value is:

(1) $$P=1-\overset{m-1}{\underset{i=0}{\sum }}\frac{\left(\begin{array}{c} M\\ i \end{array}\right)\left(\begin{array}{c} N-M\\ n-i \end{array}\right)}{\left(\begin{array}{c} N\\ n \end{array}\right)}$$

where *N* is the number of all genes with GO or KEGG annotation; *n* is the number of DEmiRNAs target genes in *N*; *M* is the number of all genes that are annotated to certain GO terms or specific pathways; and m is the number of DEmiRNAs target genes in M. GO terms or pathways meeting this condition with *p* < 0.05 were defined as significantly enriched GO terms or pathways in the DEmiRNAs target genes. The column chart of GO enrichment and the bubble diagram of KEGG pathway enrichment were drawn using the ggplot2 package (R package). The interactive network between differentially expressed miRNAs and their targeted mRNA, related to cell cycle and inflammation, was visualized in Cytoscape (https://cytoscape.org/release_notes_3_6_0.html#manual, version 3.6.0 accessed on 16 November 2017).

### 2.6. Quantitative PCR

The quantity and integrity of the total RNA of the spleen tissues after microRNA sequencing were tested [22]. The reverse transcription of qualified RNA (500 ng for each sample) and real-time quantitative PCR proceeded according to our previous work [23]. The primers shown in Table S1 were designed with Premier 5.0 software and synthesized by the Shanghai Bioengineering Co., Ltd. (Songjiang, Shanghai, China). The specificity analysis of primers was performed preliminarily by using the Primer-Blast software. The relative quantification of gene expression was calculated as a ratio of the target gene to the housekeeping gene by the 2^−ΔΔCt^ method [24] under the premise of the same amplification efficiency of the target gene and the housekeeping gene. In this study, we chose GAPDH as the housekeeping gene from three candidate genes, GAPDH, β-actin and hydroxymethylbilane synthase, which was evaluated by the BestKeeper software (version 1) to be steadily expressed (coefficiency of correlation = 0.955) in spleen tissues.

### 2.7. Statistical Analysis

All the data were analyzed by a one-way analysis of variance using SPSS 19.0 (SPSS Inc., Chicago, IL, USA). The statistical model included dietary treatment as a fixed effect. Data are expressed as means ± SD. Statistical significance was declared at *p* < 0.05. The *p* values between 0.05 and 0.10 were considered as trending toward significance.

## 3. Results

### 3.1. Visceral Organ Development and LPS Concentration

The total weight and organ indexes of the heart and spleen and kidney weight increased with the HC diet (*p* < 0.05, Table 2). Meanwhile, the lung weight tended to increase with the HC diet (*p* < 0.1). However, the liver weight and relative weight were not affected by the HC diet (*p* > 0.05). Additionally, LPS (lipopolysaccharide) concentration in the liver decreased with the HC diet (*p* < 0.05, Table 3); however, blood LPS concentration was not affected by the HC diet (*p* > 0.05). The splenic LPS concentration was not changed by the increased concentrate ratio in diets (*p* > 0.05).

### 3.2. Overview of the Deep-Sequencing Data

**Figure 1:**
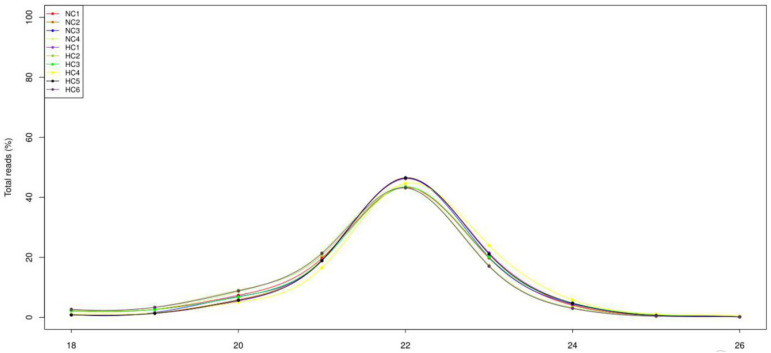
Size distribution of sequenced small RNAs from the NC group and HC group. The majority of the reads were 22 nt in length.

After high-throughput sequencing, a total of 12.4 Mb; 15.2 Mb; 14.2 Mb; and 13.7 Mb raw reads, and 18.3 Mb; 10.9 Mb; 14.2 Mb; 10.9 Mb; 11.5 Mb; and 10.0 Mb raw reads were obtained in the NC and HC groups, respectively. After removing the junk sequences, sequences with a length of more than 27 nt or less than 17 nt, RNA family sequences (Figure S1) and repeat sequences, 5.5 Mb; 10.1 Mb; 9.14 Mb; 6.46 Mb and 12.1 Mb; 4.33 Mb; 4.87 Mb; 6.74 Mb; 7.89 Mb; and 3.97 Mb high-quality reads, respectively, remained for further analysis (Table S2). After filtering the read length and analyzing the length distribution, it transpired that most of the reads were distributed between 20 and 23 nt, with 22 nt being the most abundant (Figure 1).

### 3.3. Differentially Expressed microRNAs, Gene Function, Pathway and Interactive Network

**Figure 2:**
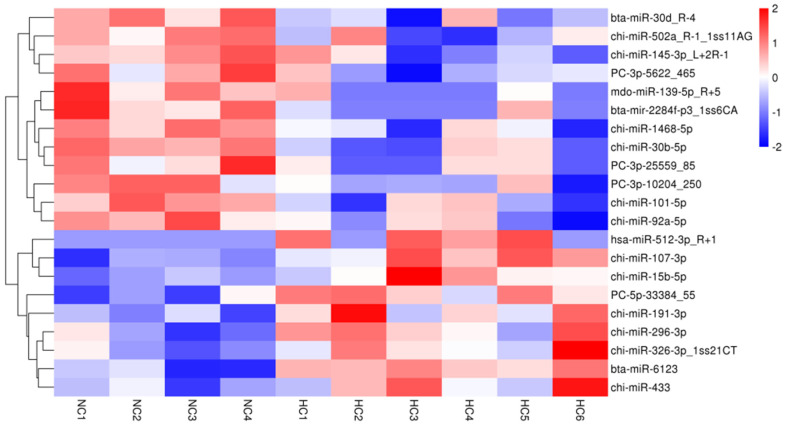
A heatmap of differentially expressed miRNAs in the NC and HC diets with a fold-change > 2.0 and *p* < 0.05.

**Figure 3:**
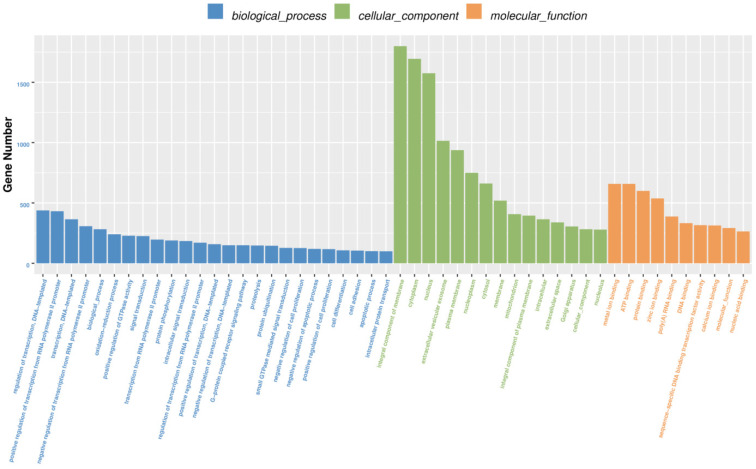
The GO enrichment analysis of the differentially expressed miRNA target genes in the spleen tissues.

**Figure 4:**
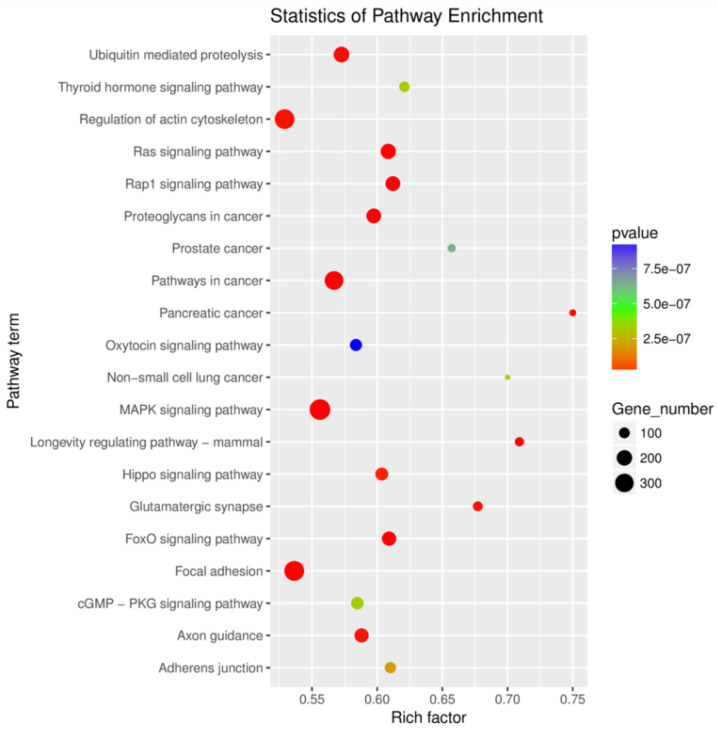
The top 20 enriched KEGG terms of the differentially expressed microRNAs target genes in the spleen tissues.

**Figure 5:**
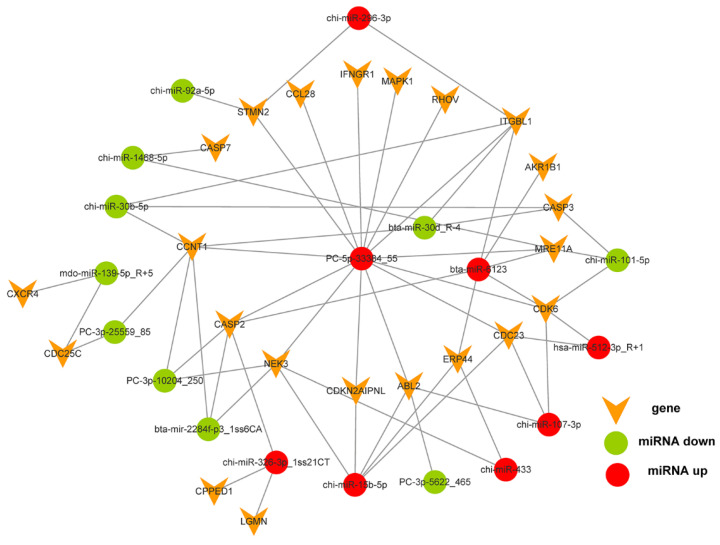
The interactive network between the differentially expressed microRNAs associated with cell cycle and inflammation, and their potential target genes.

Of the 596 expressed miRNAs, twenty-one were differentially expressed, with nine upregulated and twelve downregulated. All DEmiRNAs showed a more than 2.0-fold change threshold and a *p*-value of less than 0.05 (Figure 2 and Table S3). To explore the function of our aberrantly expressed miRNAs, the Gene Ontology (GO) consortium and Kyoto Encyclopedia of Genes and Genomes (KEGG) pathways for annotation analysis were further performed. Based on the GO enrichment analysis (*p*-value of Fisher’s exact test < 0.01), these differentially expressed miRNAs were found to be involved in the regulation of transcription, the oxidation-reduction process, signal transduction, protein phosphorylation, ATP binding, metal ion binding, protein binding, zinc ion binding and poly(A) RNA binding (Figure 3). Functional pathway analysis showed that the MAPK, Ras, Rap1, FOXO signaling pathway, and focal adhesion were significantly enriched by the HC diet (Figure 4, Table S4). The interactive network of miRNAs associated with cell cycle and inflammation and the corresponding targeted genes is shown in Figure 5. It appears that miR-30d, miR-6123 and mi-15b play an important role in regulating several genes in this network.

### 3.4. Targeted Gene Expression

To check the changes in the target mRNA of differentially expressed microRNAs, we performed a real-time quantitative PCR. Twenty-four target genes were selected. As a result, transcripts of caspase 7 (*CASP7*), cyclin T1 (*CCNT1*), cell division cycle 14A (*CDC14A*), cell division cycle 23 (*CDC23*), cyclin-dependent kinase 6 (*CDK6*), interferon-gamma receptor 1 (*IFNGR1*) and calcineurin-like phosphoesterase domain-containing 1 (*CPPED1*) were downregulated by the HC diet (*p* < 0.05, Table 4). The expression of chemokine (C-C motif) ligand 28 (*CCL28*), MRE11 homolog A, and double-strand break repair nuclease (*MRE11A*) tended to be downregulated by the HC diet, while the Ras homolog family member V (*RHOV*) expression tended to be upregulated (*p* < 0.1). Furthermore, the expression of the remaining genes was not affected by the HC diet (*p* > 0.05).

## 4. Discussion

Previous researchers have reported that feeding with an HC diet led to the translocation of LPS from the digestive system into the circulating blood [13]. It has been reported that LPS entered the liver and caused inflammation [16]. As one of the portal-drained viscera, LPS can also enter the spleen via the splenic artery. Therefore, we initially determined the LPS concentrations in the blood, liver and spleen. The results indicated that the HC diet decreased the LPS level in the liver without changes in the blood and spleen, which finding was not consistent with the previous study [16]. The observed lack of changes in LPS concentrations in the blood and spleen reflected that LPS, generated in the rumen, could be translocated into the systemic circulation, and an impairment of the barrier function of the fore-stomach epithelium probably contributed to this translocation [25,26]. Extra LPS in the blood might be cleared from the portal circulation by the liver [16,27], a process that involves macrophages or neutralization by lipoproteins [28]. The lower LPS in the livers of goats fed with the HC diet and the increment of the liver mass revealed that the liver clearance of LPS was enhanced. The increased spleen mass may suggest greater blood volume, altered red blood cell dynamics, or an immune response in goats fed with the HC diet. We speculated that some microRNAs might be involved in the regulation of spleen function. Therefore, the microRNA profile was further determined by Solexa sequencing.

As we expected, a total of 21 microRNAs were differentially expressed in the spleens of goats fed with the HC diet. Except for miR-512, miR-51b, miR-6123, miR-139, and miR-2284f, other microRNAs were conserved in *Capra hircus*. Further functional analysis showed that target genes of these microRNAs were involved in 124 pathways, of which MAPK, Ras, Rap1, FOXO signaling pathway, and focal adhesion were the most enriched ones. According to the dbDEMC2.0 database, miR-107 and miR-191 expressions were downregulated in the splenic lymphoma of humans, while the miR-92 and miR-30b expressions were upregulated [29]. However, in the present study, these four microRNAs showed an opposite trend in the spleen of goats fed with the HC diet. This result indicated that these four microRNAs could act as the surveillance molecules for splenic lymphoma. Furthermore, miR-107 expression was significantly upregulated, accompanying a decline in *CDK6* and *CDC23* mRNA expression in the spleen. It has been reported that miR-107 plays a role in increasing macrophage adhesion and regulating adipocyte differentiation and lipid storage by inhibiting *CDK6* expression [30,31]. An inverse correlation between miR-107 and *CDK6* was confirmed in our study; whether *CDC23* expression is negatively related to miR-107 expression needs to be explored in future studies.

Feeding the HC diet also upregulated splenic miR-326 expression. According to the interactive network results, its target gene *CPPED1* was downregulated at the mRNA level. It has been proven that miR-326 interacts with certain long noncoding RNAs, such as the testis development-related gene 1 (TDRG1) and SNHG1, and acts by promoting cell proliferation, migration and invasion [32,33]. miR-326 also regulated interleukin 17-producing T helper cell differentiation [34]. Therefore, our result indicated that miR-326 was probably involved in promoting splenocyte proliferation through the repression of *CPPED1*gene expression.

In one study, miR-512-3p participated in the human trophoblast function by targeting phosphatase 3, regulatory subunit B, alpha (PPP3R1) [35]. It also suppressed tumor growth by targeting human telomerase reverse transcriptase in head and neck squamous cell carcinoma [36]. The expression of miR-512-3p in the spleen was upregulated by the HC diet, which was concomitant with a reduction in *CDK6* (the target gene) mRNA expression. The upregulation of miR-512-3p would boost the proliferation of splenocytes in goats via inhibiting *CDK6* expression. Furthermore, miR-15b-5p expression was also stimulated by the HC diet and its target gene *CDC23* mRNA was suppressed. It has been reported that miR-15b-5p ameliorated high glucose-induced kidney podocyte injury through repressing apoptosis, oxidative stress and inflammatory responses [37]. However, it can also induce mesangial cell apoptosis by targeting *BCL2* under high glucose, predicting kidney injury in diabetic nephropathy [38]. Therefore, miR-15b-5p stimulation in the spleen further inferred its function in abating splenocyte apoptosis.

In a previous study, miR-296-3p functioned as a tumor suppressor in non-small-cell lung cancer [39] and sinonasal inverted papilloma [40]. In another, miR-191 has been identified as an important oncogenic miRNA in breast, colon, lung, pancreas and stomach cancers [41,42,43,44]. Its function was estrogen-dependent. miR-433 was also a tumor suppressor in breast cancer and oral squamous cell carcinoma [45,46]. Its expression markedly suppressed cell proliferation, invasion and migration by targeting HDAC6 [47]. In the present study, the upregulation of miR-296-3p, miR-191, and miR-433 expression by the HC diet indicated that the HC diet would prevent the spleen from developing tumors. Additionally, our study first observed miR-6123 expression in the spleen of goats, although it was conserved in *Bos taurus.* Its physiological function needs further investigation.

The miRNA-30 family includes miR-30b and miR-30d, together with miR-30a, miR-30c, and miR-30e. miR-30b could regulate interleukin-10 and toll-like receptor 4 expressions in T-lymphocytes [48], may participate in NLRP3 inflammasome expression in chronic liver injury [49] and be involved in the inflammatory response of acute lung injury in children [50]. Meanwhile, miR-30d modulates the survival programs of neural cells by regulating autophagy and apoptosis [51]. In this study, miR-30b and miR-30d were significantly downregulated by the HC diet, demonstrating that the occurrence of splenocyte injury was very small. The genes *CASP3* and *CCNT1* were the common target genes of miR-30b and miR-30d. *CASP3*, an important element in the MAPK pathway, is necessary for apoptosis-associated proteolysis, nuclear condensation, chromatin margination and DNA fragmentation in cell-free extracts [52]. Except for the upregulation of cell proliferation, *CCNT1* expression in mature macrophages could be reinduced by activation with LPS, inferring its role in the innate immune response [53,54]. A reduction in *CCNT1,* rather than *CASP3* expression, was observed with the HC diet, displaying that the proliferation of splenocytes was enhanced by the HC diet.

It has been reported that miR-1468-5p inhibits human glioma cell proliferation and induces cell cycle arrest by targeting the ribonucleotide reductase large subunit M1 [55]. Based on bioinformatic analyses, *CASP7* is predicted to be a miR-1468-5p target. Activated *CASP7* and endoplasmic reticulum stress were induced by cytolytic T cell-delivered granulysin in a whole-cell system [56]. It has been documented that *CASP7* deficiency protected against LPS-induced lymphocyte apoptosis and improved endotoxemia-associated mortality [57]. A decline in *CASP7* mRNA in the spleens from the HC diet group was observed in this study, inferring that an HC diet could inhibit the endoplasmic reticulum stress induced by splenocyte apoptosis. However, a synchronous decrease in miR-1468-5p and *CASP7* expression was observed in the spleens of goats fed with the HC diet. We speculate that miR-1468-5p participated in the regulation of the cell cycle in splenocytes by targeting other genes rather than *CASP7*.

It has been reported that miR-502a inhibited cell proliferation in esophageal squamous cell carcinoma [58]. miR-145 was reported to regulate IL-10 expression by targeting histone deacetylase 11 and promoted activated macrophage (M2) polarization. miR-145 also regulated M2 macrophage polarization by targeting IL-16 and enhancing IL-10 expression [59]. The deletion of miR-139-5p activated MAPK, NF-κB and STAT3 signaling, and promoted intestinal inflammation [60]. Splenic miR-502a expression was inhibited by the HC diet, indicating that the splenocyte proliferation was enhanced. However, the reduction in miR-145 and miR-139-5p expression by the HC diet meant the antimicrobial function of the spleen might be weakened by feeding with an HC diet.

The miR-2284 family was highly expressed in bovine mammary epithelial cells [61] and was predicted to be involved in regulating the expression of casein genes (*CSN1S1* and *CSN2*) in the bovine mammary gland [62]. miR-2284x is significantly higher in bovine whey than in porcine whey, and its level is significantly higher in the bovine milk-feeding piglets than those in the porcine milk-feeding piglets on the first two weeks [63]. miR-101 can suppress tissue fibrosis [64,65]. miR-92a has been shown to be associated with immune response and virus-host interactions [66]. miR-92a also induced apoptosis in the WP of spleens in mice [67]. The downregulation of miR-2284f, miR-101 and miR-92a induced by the HC diet indicated that splenocyte apoptosis was inhibited by the HC diet and spleen fibrosis may be repressed.

IFN-γ exerted its antiviral activity through *IFNGR1* (the alpha-chain) and *IFNGR2* (the beta-chain). Human *IFNGR* expression was restricted to the B-cell areas of adult and fetal spleens [68]. IFNGR1 protein was involved in the activating of resting T lymphocytes and preventing the reactivated lymphoblasts from experiencing apoptosis [69]. In goats, the IFNGR protein might act as a critical targeted molecule to coordinate the immune endocrine regulation of IFN-γ and neuro-regulation of autonomic nerves in the target organs, such as the gastrointestinal tract and cardiovascular system [70,71,72]. In our study, splenic *IFNGR1* mRNA expression was downregulated by the HC diet, suggesting that the HC diet would suppress the activation of resting T lymphocytes and the immune endocrine regulation of IFN-γ in the spleen. Furthermore, it has been reported that miR-378 decreased the expression of IFNGR1 protein but not its mRNA at the different stages of bovine corpus luteum development [73]. Although miR-378 expression tended to be upregulated in the spleens of goats fed with the HC diet (fold-change = 1.24, *p*-value = 0.072), whether *IFNGR1* mRNA expression is regulated by the miR-378 in goats’ spleens needs further investigation.

## 5. Conclusions

In this study, twenty-one differentially expressed miRNAs in the spleens of goats fed an HC diet were identified and functionally annotated. The interactive connection of these miRNAs and the corresponding targeted genes related to the cell cycle and inflammation were also displayed. Our results indicated that the HC diet promoted the growth of the heart and spleen by increasing their weight and organ index. The miRNA data indicated that, under the conditions of HC diet feeding, miR-326, miR-512-3p, miR-30b, miR-30d, miR-502a and their target genes’ (CPPED1, CDK6, CCNT1) expression may play an important role in the enhancement of the proliferation of splenocytes, while miR-15b-5p, miR-1468 and miR-92a probably contribute to the suppression of splenocyte apoptosis. This work could help to deepen our understanding of miRNA-level molecular events in the peripheral immune organs in mammals.

## Figures and Tables

**Table 1:** Ingredients and nutrient levels of the experimental diets (DM basis).

Item	NC	HC
Ingredient composition (%)		
Rice straw	45.0	10.0
Rice with shell	33.2	54.3
Soybean meal	9.60	15.7
Wheat bran	6.00	9.80
Fat powder	3.20	5.20
Calcium carbonate	0.50	0.80
Calcium bicarbonate	1.10	1.80
Sodium chloride	0.60	1.00
Premix ^a^	1.00	1.40
Nutrient levels ^b^, % of DM		
Crude protein	13.5	17.6
Crude ash	9.34	9.12
Crude fat	12.0	11.9
Neutral detergent fiber	49.8	38.4
Acid detergent fiber	36.5	9.51
NFC ^c^	5.74	12.5
DMI ^d^, g/d	572	602

NC, normal concentrate diet; HC, high concentrate diet. ^a^ Premix composition per kg diet: 68 mgFeSO_4_·H_2_O, 44 mg CuSO_4_·5H_2_O, 411 µg CoCl_2_·6H_2_O, 1.70 mg KIO_3_, 211 mg MnSO_4_·H_2_O, 126 mg ZnSO_4_·H_2_O, 56 µg Na_2_SeO_3_, 462 mg MgSO_4_·7H_2_O, 737 IU vitamin A, 8.29 mg vitamin E, 4.0 g NaHCO_3_, 5.1 g carrier zeolite powder. ^b^ Nutrient levels were measured values. ^c^ NFC: non-fibrous carbohydrate. NFC was calculated in accordance with NFC = DM − (CP + EE + Ash + NDF). ^d^ DMI: Dry matter intake. Data were published by Wang et al. (2019).

**Table 2:** Visceral organ weight and index (OI) and average daily gain (ADG).

Item	NC	HC	*p*-Values
Heart, g	64.5 ± 3.80	77.5 ± 6.12	0.002
Liver, g	293 ± 50.9	343 ± 57.4	0.142
Spleen, g	20.5 ± 3.30	27.0 ± 4.75	0.020
Lung, g	215 ± 36.1	260 ± 43.1	0.078
Kidney, g	54.7 ± 4.61	62.1 ± 4.18	0.016
Heart OI, % BW	0.383 ± 0.034	0.426 ± 0.012	0.028
Liver OI, % BW	1.74 ± 0.37	1.67 ± 0.038	0.652
Spleen OI, % BW	0.112 ± 0.007	0.145 ±0.026	0.023
Lung OI, % BW	1.29 ± 0.31	1.47 ± 0.089	0.256
Kidney OI, % BW	0.326 ± 0.038	0.333 ± 0.018	0.667
ADG ^a^, g/d	67.1± 19.2	125 ± 31.9	0.006

NC, normal concentrate diet; HC, high concentrate diet. ^a^ Data were published by Wang et al. (2019).

**Table 3:** Lipopolysaccharide concentrations in the blood, spleen and liver.

Item	NC	HC	*p-*Values
Blood ^a^, EU/mL	1.84 ± 0.115	1.77 ± 0.143	0.520
Spleen, ng/mgprot	180 ± 42.4	176 ± 38.0	0.889
Liver, ng/mgprot	135 ± 34.7	87.5 ± 6.27	0.015

NC, normal concentrate diet; HC, high-concentrate diet. ^a^ Data were cited by Wang et al. (2019).

**Table 4:** Expression of genes associated with cell cycle and inflammation in the spleen tissues.

Items	NC	HC	*p* Values
**Cell Cycle**			
*ABL2*	1.04 ± 0.35	0.88 ± 0.49	0.574
*CASP2*	1.01 ± 0.14	1.25 ± 0.33	0.218
*CASP3*	1.07 ± 0.40	1.14 ± 0.52	0.82
*CASP7*	1.09 ± 0.49	0.38 ± 0.11	0.017
*CCNT1*	1.01 ± 0.14	0.51 ± 0.25	0.015
*CDC14A*	1.04 ± 0.33	0.54 ± 0.31	0.04
*CDC23*	1.02 ± 0.21	0.65 ± 0.10	0.006
*CDC25C*	1.06 ± 0.41	0.78 ± 0.15	0.156
*CDC42*	1.10 ± 0.45	0.86 ± 0.24	0.304
*CDK6*	1.02 ± 0.26	0.61 ± 0.22	0.027
*CDKN2AIPNL*	1.05 ± 0.38	0.71 ± 0.36	0.213
*ITGBL1*	1.06 ± 0.43	0.75 ± 0.24	0.178
*MAPK1*	1.04 ± 0.32	0.69 ± 0.33	0.135
*MRE11A*	1.08 ± 0.48	0.65 ± 0.24	0.099
*NEK3*	1.02 ± 0.26	0.96 ± 0.32	0.746
*RHOV*	1.14 ± 0.63	2.32 ± 1.05	0.091
*STMN2*	1.01 ± 0.15	1.00 ± 0.29	0.954
**Inflammation**			
*CCL28*	1.03 ± 0.31	0.71 ± 0.23	0.089
*CXCR4*	1.06 ± 0.43	0.82 ± 0.17	0.246
*IFNGR1*	1.03 ± 0.30	0.47 ± 0.24	0.016
**Other Genes**			
*AKR1B1*	1.01 ± 0.35	0.88 ± 0.49	0.574
*LGMN*	1.07 ± 0.43	0.95 ± 0.17	0.553
*CPPED1*	1.03 ± 0.29	0.42 ± 0.18	0.006
*ERP44*	1.01 ± 0.18	0.88 ± 0.28	0.425

Legend: NC, normal concentrate diet; HC, high-concentrate diet.

## Data Availability

All the sequencing data were deposited in the publicly available NCBI’s Sequence Read Archive (https://www.ncbi.nlm.nih.gov/sra, accessed on 4 November 1988). The data are accessible through accession number PRJNA717117 (https://www.ncbi.nlm.nih.gov/sra/PRJNA717117, accessed on 4 November 1988).

## Supplementary Materials

The following are available online at https://www.mdpi.com/article/10.3390/biology10090832/s1, Figure S1: Small RNA reads from NC group and HC group were BLASTed against the Rfam database non-coding RNA to annotate rRNA, snRNA, tRNA, snoRNA and others RNAs, Table S1: Information of primers used in the real-time quantitative PCR, Table S2: Overview of reads from raw data to cleaned sequences of small RNA sequences, Table S3: The differentially expressed miRNAs between NC group and HC group, Table S4: The top20 enriched KEGG terms of the differentially expressed microRNAs in the spleen tissues.
