# Supplementary material for: The Regulatory Mechanism of Feeding a Diet High in Rice Grain on the Growth and microRNA Expression Profiles of the Spleen, Taking Goats as an Artiodactyl Model
Source: Biology (Basel). 2021 Aug 26;10(9):832. doi: 10.3390/biology10090832 (PMC8467863; doi:10.3390/biology10090832)
Supplement: Supplementary file 1 [file biology-10-00832-s001.zip › Supplemental figure1.pdf]

Figure S1 Small RNA reads from NC group and HC group were blasted against the Rfam database non-coding RNA to annotate rRNA, snRNA, tRNA, snoRNA and others RNAs.

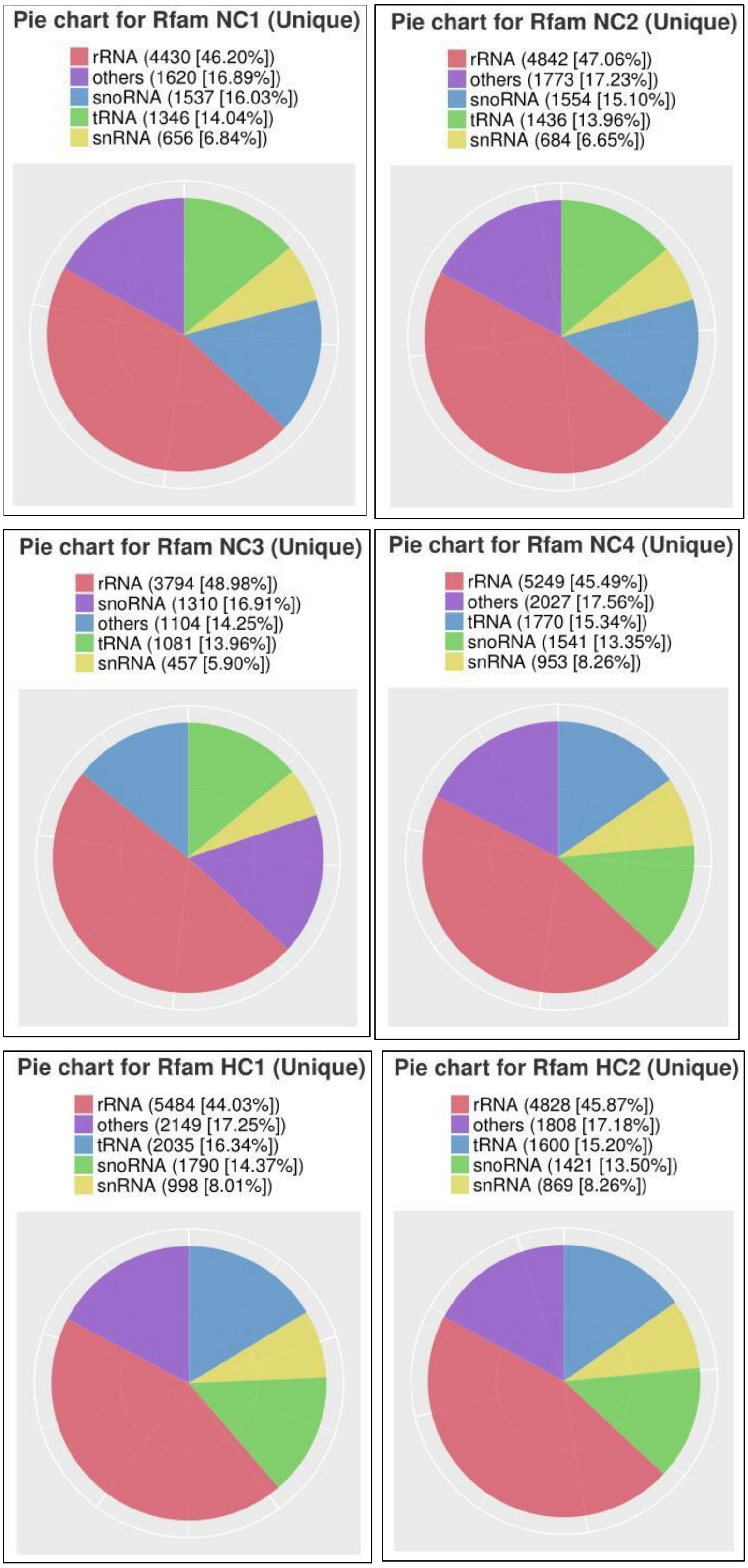

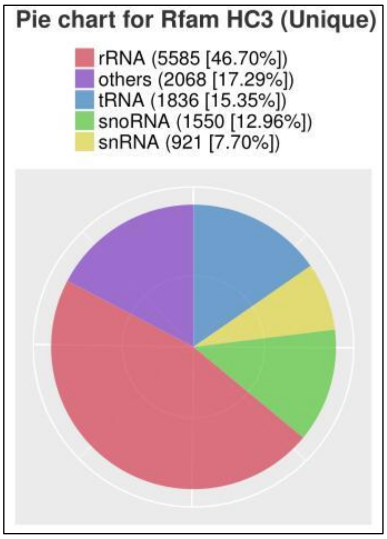
Pie chart for Rfam HC3 (Unique)
rRNA (5585 [46.70%])
others (2068 [17.29%])
tRNA (1836 [15.35%])
snoRNA (1550 [12.96%])
snRNA (921 [7.70%])

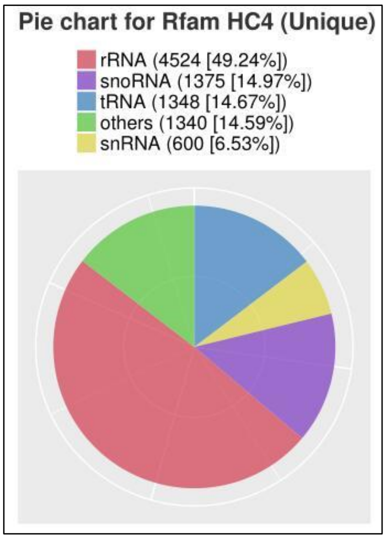
Pie chart for Rfam HC4 (Unique)
rRNA (4524 [49.24%])
snoRNA (1375 [14.97%])
tRNA (1348 [14.67%])
others (1340 [14.59%])
snRNA (600 [6.53%])

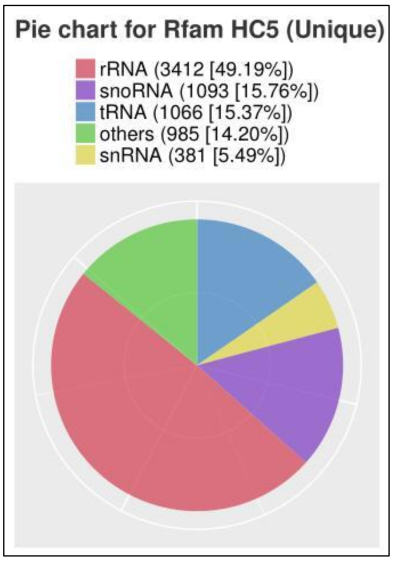
Pie chart for Rfam HC5 (Unique)
rRNA (3412 [49.19%])
snoRNA (1093 [15.76%])
tRNA (1066 [15.37%])
others (985 [14.20%])
snRNA (381 [5.49%])

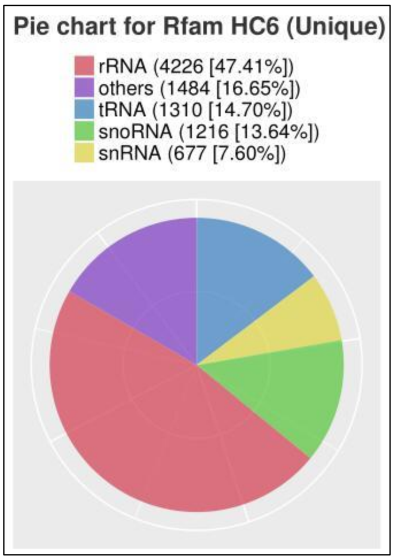
Pie chart for Rfam HC6 (Unique)
rRNA (4226 [47.41%])
others (1484 [16.65%])
tRNA (1310 [14.70%])
snoRNA (1216 [13.64%])
snRNA (677 [7.60%])
